# Effectiveness of transcranial direct current stimulation over dorsolateral prefrontal cortex in patients with prolonged disorders of consciousness: A systematic review and meta-analysis

**DOI:** 10.3389/fneur.2022.998953

**Published:** 2022-09-26

**Authors:** Siwei Liu, Qiang Gao, Min Guan, Yi Chen, Shuhai Cheng, Lin Yang, Wei Meng, Chunyan Lu, Bingqian Li

**Affiliations:** Department of Rehabilitation Medicine, West China Hospital, Sichuan University, Chengdu, China

**Keywords:** prolonged disorder of consciousness, meta-analysis, transcranial direct current stimulation, minimally conscious state, unresponsive wakefulness syndrome, neurorehabilitation, non-invasive brain stimulation

## Abstract

**Background:**

Transcranial direct current stimulation (tDCS) has been widely studied for treatment of patients with prolonged disorders of consciousness (PDOC). The dorsolateral prefrontal cortex (DLPFC) is a hot target for intervention, but some controversies remain.

**Purpose:**

This review aimed to systematically investigate the therapeutic effects of DLPFC-anodal-tDCS for patients with PDOC through a meta-analysis approach.

**Data sources:**

Searches for relevant articles available in English were conducted using EMBASE, Medline, Web of Science, EBSCO, and Cochrane Central Register of Controlled Trials from inception until March 26, 2022.

**Study selection:**

All randomized parallel or cross-over controlled trials comparing the effect of intervention with active-tDCS and Sham-tDCS on Coma Recovery Scale Revised (CRS-R) score in individuals with PDOC were included.

**Data extraction:**

Two authors independently extracted data, assessed the methodological quality, and rated each study.

**Data synthesis:**

Ten randomized parallel or cross-over controlled trials were eligible for systematic review, and eight of the studies involving 165 individuals were identified as eligible for meta-analysis. Compared with Sham-tDCS, the use of anode-tDCS over DLPFC improved the CRS-R score (SMD = 0.71; 95% CI: 0.47–0.95, *I*^2^ = 10%). Patients with PDOC classified as MCS and clinically diagnosed as CVA or TBI may benefit from anode-tDCS.

**Limitations:**

Failure to evaluate the long-term effects and lack of quantitative analysis of neurological examination are the main limitations for the application of anode-tDCS.

**Conclusions:**

Anodal-tDCS over the left DLPFC may be advantageous to the recovery of patients with MCS and clinically diagnosed with CVA or TBI. There is a lack of evidence to support the duration of the disease course will limit the performance of the treatment. Further studies are needed to explore the diversity of stimulation targets and help to improve the mesocircuit model.

**Systematic review registration:**

https://www.crd.york.ac.uk/prospero/display_record.php?RecordID=279391, identifier: CRD42022279391.

## Introduction

Disorders of consciousness (DOC) is a widespread brain dysfunction caused by direct or indirect injury to the neural network, which regulates the level of arousal and/or awareness (1). The common causes of DOC include traumatic brain injury (TBI), cerebrovascular accident (CVA), and hypoxic brain injury (HIBI) (2). In clinical practice, DOC with a disease course of more than 4 weeks (28 day) is considered a prolonged disorder of consciousness (PDOC) (3). Patients with PDOC are completely dependent on other people for care due to the lack of functional communication and action ability, resulting in tremendous social and economic burdens and ethical risks (4). The prevalence of DOC in Europe ranges from 0.2 to 6.1 patients per 100,000 inhabitants, which is estimated to be about 10 times that in the UK and the United States (4, 5). Paradoxically, this ratio is still progressively increasing with the development of medical technology.

PDOC can be divided into unresponsive wakefulness syndrome (UWS) and minimally conscious state (MCS) according to the degree of retention of awareness (6). UWS is also traditionally called as vegetative state (VS), and MCS can be further divided into MCS- and MCS+ (7, 8). We can use the Coma Recovery Scale Revised (CRS-R) to infer the degree of consciousness retention through the behavioral characteristics of patients and make differential diagnosis for PDOC (9, 10). According to the single score of the CRS-R subscale, UWS, MCS-, MCS+, and emergence from MCS (eMCS) can be qualitatively diagnosed. MCS is considered to have a higher level of awareness than UWS, while MCS+ is better than MCS- and eMCS is usually presented as a sign of breaking away from DOC (10, 11). The alteration of consciousness can be quantitatively evaluated and compared through the total score or the changes in CRS-R (10, 11).

According to recent research on the neuropathology of patients with PDOC, a mesocircuit model with central thalamus as the core has been gradually revealed. The anatomic structures mainly involved in mesocircuit mode include the prefrontal cortex, central thalamus, striatum, pallidum, and default mode network (DMN). The model suggests that the main pathological change in PDOC is the withdrawal of excitatory synaptic activity across the cerebrum produced by deafferentation or disfacilitation of DMN and central thalamus and striatal neurons (1, 12). Activation or inhibition of some neural structures in this mesocircuit model is supposed to be related to the therapeutic effect of PDOC (12). In the mesocircuit model, the activation of the dorsolateral prefrontal cortex (DLPFC) is supposed to induce a stronger connectivity between the prefrontal cortex and the thalamus because the prefrontal cortex has many connections with the striatum, which is conducive to releasing thalamic activity through striatal inhibition of the pallidum (1, 12). DLPFC also plays a critical role in advanced cognitive functions, such as working memory and executive control (13). Therefore, DLPFC mediation is one of the main directions in the field (14).

The only recommended treatment for PDOC by the guidelines is amantadine (Level B) (2) due to its definite effect on alleviating the tonic inhibition of pallidum to the central thalamus by striatum activation (15). However, drug resistance limits the application of this method in some individuals (1). Thus, researchers have constantly proposed and studied a variety of therapies (12). Non-invasive brain stimulation (NIBS) has high application potential and employs a safe and convenient operation. Among various NIBS types, transcranial direct current stimulation (tDCS) has been relatively more widely studied in the treatment of PDOC (16). The tDCS can apply a weak-intensity direct current *via* scalp electrodes to affect the release of neurotransmitter, induce neuroplasticity, and thus modulate neural excitability (17). Stimulating *via* the anode usually enhances the excitability, while that *via* the cathode will lead to decrease (17).

Combined with the mesocircuit model and the characteristics of tDCS, DLPFC-anodal-tDCS is considered to have a positive therapeutic value for PDOC (18). However, most correlational studies only have a relatively small sample size, and the outcomes are highly inconsistent (19). The effectiveness of tDCS on patients with UWS and MCS varies (16, 20). No meta-analysis has been published yet using DLPFC-anode-tDCS for patients with PDOC, and only one subgroup-analysis in a systematic review on the effects of all types of NIBS on patients with DOC briefly discusses this issue (21). This topic should be further discussed. Therefore, this study aimed to systematically investigate the therapeutic effects of DLPFC-anodal-tDCS on patients with PDOC through a meta-analysis approach and evaluate the potential bias and methodological limitations of the studies included in this systematic review.

## Methods

We have registered the protocol of this systematic review and meta-analysis on the international prospective register of systematic reviews (PROSPERO), and the register number is: CRD42022279391. This research follows the PRISM reporting specifications.

### Inclusion criteria

Studies that met all the following criteria were included: ① randomized parallel controlled trial or randomized cross-over controlled trial; ② the recruited participants were human patients diagnosed with PDOC; ③ the intervention to the participants was anode-tDCS over DLPFC with a sham stimulation as the control; ④ the results of CRS-R have been reported; and ⑤ written in English.

### Exclusion criteria

Studies meeting the three criteria were excluded: ① any study using tDCS combined with other treatment methods (such as drugs) as the experimental intervention; ② any study using the DLPFC combined with other brain regions as the anode-tDCS target; and ③ conference abstracts or study protocols.

### Literature search

The literature searches were performed in five electronic databases: EMBASE, MEDLINE (*via* PubMed), Web of Science, EBSCO, and Cochrane Central Register of Controlled Trials. The retrieval time limit was set from the establishment of the database to March 26, 2022, and the language was limited to English. The search string was built as follows: [(tDCS) OR (transcranial direct current stimulation)] AND [(MCS) OR (minimally conscious state) OR (disorder of consciousness) OR (coma) OR (unresponsive wakefulness syndrome) OR (vegetative state) OR (disturbance of consciousness)]. The examples of specific search strategies are presented in Appendix.

### Data extraction

Two authors preliminarily excluded irrelevant papers *via* the title, abstract, and publication type of each article. Two other authors read all the full texts and completed data extraction independently. Any disagreement was resolved by discussion until consensus was reached or by consulting a third author. The following data were extracted: author, study design, year of publication, and the country where the study was conducted; sample size, demographic characteristics, disease course, and diagnosis; protocol of tDCS (including stimulation target, intensity, duration of single intervention season, duration of washout period, total intervention period, design of sham stimulation, etc.), and CRS-R scores before and after the experimental intervention. According to the data extraction protocol, ineligible articles were excluded.

### Risk of bias and quality assessment

The risk of bias of the included studies was assessed *via* the PEDro scale for methodological quality (22). The scale comprises 11 items with a total score of 0–10 by adding the ratings of items 2–11. Higher scores indicate superior methodological quality. Scores less than 4 are “poor,” 4–5 are “fair,” 6–8 are “good,” and 9–10 are “excellent” (22). And we used the *Cochrane risk of bias tool* to assess each study which classifies the low, high, or unclear risk of bias based on the following items: random sequence generation, allocation concealment, blinding of participants and personnel, blinding of outcome assessment, incomplete outcome data, selective reporting, and other biases (23). We also used the Grading of Recommendations Assessment, Development and Evaluation (GRADE) to assess the certainty of evidence.

The same two authors who completed the data extraction independently rated each study. Divergences were also resolved by discussion or by judgement of the third author.

### Data synthesis and analysis

Statistical analyses were conducted using Review Manager V5.4 (Cochrane Collaboration, Copenhagen, Denmark), and publication bias was tested by Stata V16.0. Before pooled estimation, we first removed the data of participants who did not meet the PDOC diagnostic criteria (disease course less than 28 days or diagnosed as eMCS) from the obtained datasets (24, 25). The effect size of the outcome measures was identified by giving the standardized mean difference (SMD) with a 95% confidence interval (CI) in the change in CRS-R between the baseline and the end of interventions. Authors would be contacted *via* email for missing data.

Heterogeneity was tested using the *Chi*^2^ test and *I*^2^ index. The extent of heterogeneity was estimated as follows: low (25%), moderate (50%), and high (75%) *I*^2^-values (26). If the *P*>0.1 and the *I*^2^ < 50%, then the fixed-effect meta-analysis would be conducted; otherwise, when the *P* ≤ 0.1 or the *I*^2^ ≥ 50%, the random-effect model would be used. In addition, we used subgroup analysis to assess the influence of patients with different disease courses or diagnoses (including the differences in diagnosis of PDOC and clinical diagnosis). Sensitivity analysis using the leave-one-out method would be conducted in the meta-analysis of all studies and when there was high heterogeneity in any subgroup analysis. Funnel plot and Egger's test were used to assess publication bias.

## Results

### Study screening

**Figure 1:**
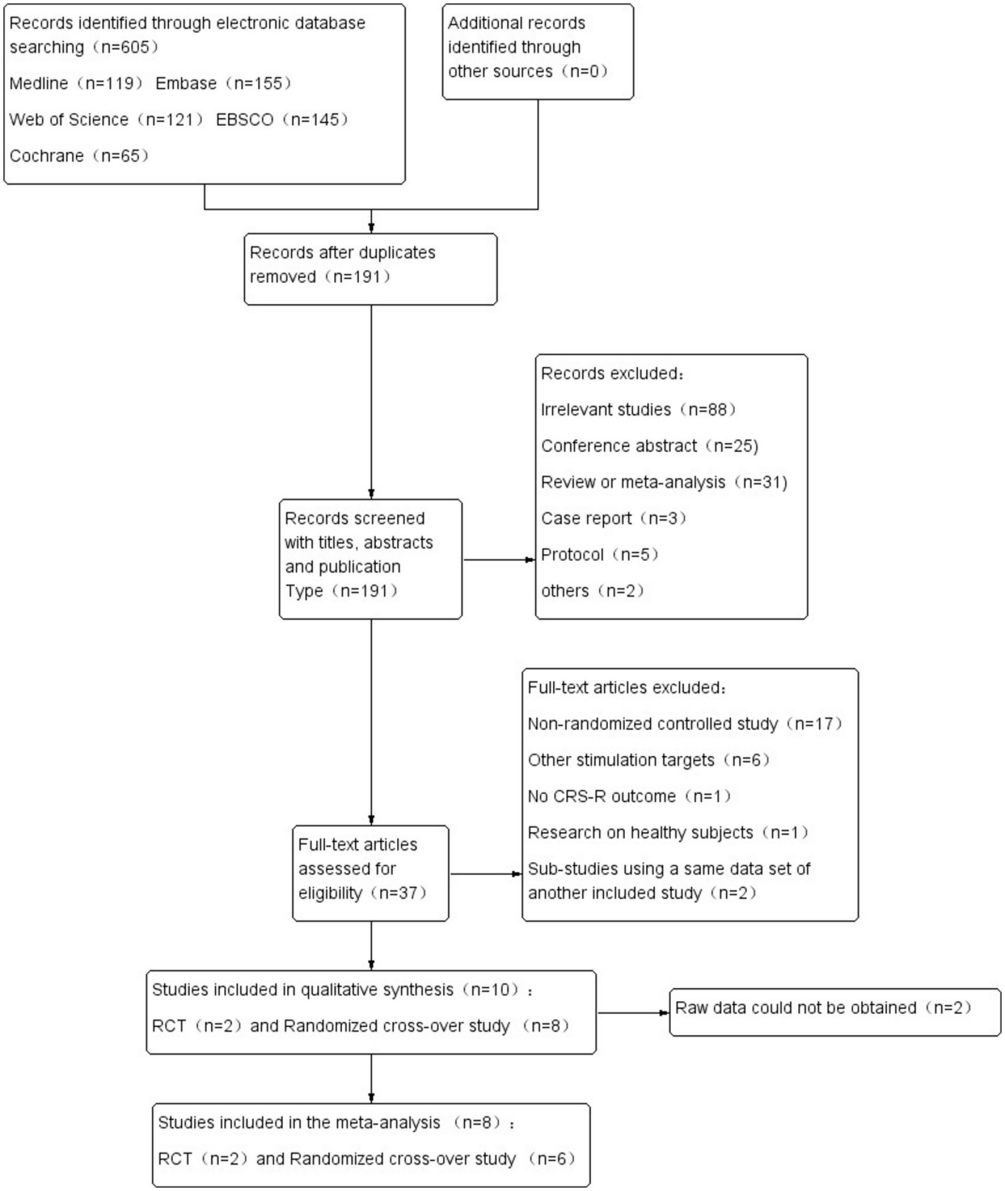
Study flow diagram.

The process of literature screening is shown in Figure 1. The original search found 605 records. After removing the duplicates, 191 records remained. After the preliminary screening of the titles, abstracts, and article types, 37 were left. In data extraction, we excluded 27 studies based on our inclusion and exclusion criteria. Ten studies were used in our review, but two of them were unable to obtain the raw data (27, 28). Thus, only eight studies were finally included in quantitative synthesis, involving a total of 289 pieces of data (active-tDCS and sham-tDCS: 147 and 142, respectively) (24, 25, 29–34). Due to the existence of cross-over trials, the actual participants totaled 165. The details and characteristics of the included studies are shown in Table 1.

**Table 1:** The characteristics of included studies.

**References**	**Country**	**Study design**	**Participants^a^**	**Clinical diagnoses**	**Disease course (month^b^)**	**Intervention protocol**	**Duration of washout**	**Outcome measurement**	**PEDro Scale**	**Raw data available**
Thibaut et al. (24)	Belgium	randomized controlled cross-over trial	*n* = 50 UWS/MCS = 23/27	TBI/HIBI/CVA 27/13/10	16.00 (2.75, 44.50)	L-DLPFC 2mA anodal tDCS, 20 min	2 days	CRS-R	8	Yes
Thibaut et al. (30)	Belgium	randomized controlled cross-over trial	*n* = 16 MCS only	TBI/HIBI/CVA 11/3/2	21.00 (17.00, 122.50)	L-DLPFC 2 mA anodal tDCS, 20 min/qd, 5 days	7 days	CRS-R	7	Yes
Zhang et al. (31)	China	randomized controlled parallel trial	Active (*n* = 13): UWS/MCS = 5/8	TBI/HIBI/CVA 5/2/6	4.80 (2.15, 8.25)	L-DLPFC 1–2 mA anodal tDCS, 20 min/bid, 2 weeks	Not applicable	CRS-R, ERP	7	Yes
			Sham (*n* = 13): UWS/MCS = 6/7	TBI/HIBI/CVA 7/3/3	3.40 (2.15, 6.90)					
Estraneo et al. (32)	Italy	randomized controlled cross-over trial	*n* = 13 UWS/MCS = 7/6	TBI/HIBI/CVA 1/6/6	10.00 (4.50, 26.00)	L-DLPFC 2 mA anodal tDCS, 20 min/qd, 5 days	7 days	CRS-R, EEG	7	Yes
Martens et al. (33)	Belgium	randomized controlled cross-over trial	*n* = 27 MCS only	TBI/HIBI/CVA 12/10/5	88.00 (35.00, 149.00)	L-DLPFC 2 mA anodal tDCS, 20 min/qd, 4 weeks	56 days	CRS-R	8	Yes
Wu et al. (34)	China	randomized controlled parallel trial	Group A (*n* = 5): UWS/MCS = 2/3	TBI/HIBI/CVA 2/0/3	168.00 (55.00, 242.00)	L-DLPFC 2 mA anodal tDCS, 20 min/qd, 2 weeks	Not applicable	CRS-R, EEG	7	Yes
			Group B (*n* = 5): UWS/MCS = 4/1	TBI/HIBI/CVA 1/0/4	158.00 (65.50, 425.00)	R-DLPFC				
			Sham (*n* = 5): UWS/MCS = 2/3	TBI/HIBI/CVA 2/3/0	55.00 (37.50, 130.50)	L-DLPFC				
Carrière et al. (29)	Belgium	randomized controlled cross-over trial	*n* = 9 MCS only	TBI/HIBI/CVA/other 3/2/3/1	5.00 (3.50, 12.00)	L-DLPFC 2 mA anodal tDCS, 20 min	2 days	CRS-R, EEG	6	Yes
Barra et al. (25)	Belgium	randomized controlled cross-over trial	*n* = 9 MCS only	TBI/HIBI/CVA 2/1/6	4.00 (2.75, 5.40)	L-DLPFC 2 mA anodal tDCS, 20 min	>5 days	CRS-R, EEG	8	Yes
Cavinato et al. (27)	Italy	randomized controlled cross-over trial	*n* = 24 UWS/MCS = 12/12	TBI/HIBI/CVA 9/8/7	25.00 (8.25, 69.00)	L-DLPFC 2 mA anodal tDCS, 20 min/qd, 2 weeks	10 days	CRS-R, EEG	7	No
Thibaut et al. (28)	Belgium	randomized controlled cross-over trial	*n* = 13 UWS/MCS = 6/7	TBI/HIBI/CVA 6/3/4	12.06 (8.10, 44.85)	B-DLPFC 2 mA anodal tDCS, 20 min	>2 days	CRS-R, EEG	8	No

MCS, minimally conscious state; UWS, unresponsive wakefulness syndrome; TBI, traumatic brain injury; HIBI, hypoxic ischemic brain injury; CVA, cerebrovascular accident; L/R/B-DLPFC, left/right/bilateral-dorsolateral prefrontal cortex; tDCS, transcranial direct current stimulation; CRS-R, Coma Recovery Scale-Revised; EEG, electroencephalogram; ERP, event-related potential.

^a^The data of participants who do not meet the PDOC diagnosis has been eliminated from the total number of participants according to the details of the raw data.

^b^The duration of the disease course were expressed by the median (25 and 75% quartile).

### Methodological quality

Quantitative quality analysis using the PEDro scale illustrated that the 10 trials were all of “good” methodological quality, as each individual trial had scores between 6 and 8. The mean PEDro score of all included trials was 7.30 ± 0.67 (Supplementary Table S1).

The risk of bias summarized with the *Cochrane risk of bias tool* is shown in Supplementary Figures S1, S2. The methods of randomization or random sequence generation were not clearly described in 4 of the 10 studies (29, 31, 32, 34), and the allocation concealment does not make clearly in 6 of them (27–29, 31, 32, 34). The evaluators and participants were blinded in 9 of the 10 studies except for 1 study that did not report blinding of outcome assessment (28). Incomplete data in 5 studies were due to withdrawal of the participants (25, 27, 29, 30, 33); despite reasonable explanation, the rates of drop-out were high in 3 studies (23–30%) (29, 30, 33). With regard to other bias, in 1 RCT study (34), there was a high heterogeneity in the baseline of clinical diagnosis between the two groups, although they claimed that the study was randomized; and for the 8 cross-over trials, 1 study considered that it failed to avoid the carry-over effect (30), and 3 of them (27, 28, 32) failed to discuss the carry-over effect.

On the basis of the GRADE, the quality of evidence for the DLPFC-anode-tDCS was classified as moderate. The result is presented in Supplementary Table S2.

### Meta-analysis—effects of DLPFC-anode-tDCS on PDOC

In almost all included studies, left DLPFC (LDLPFC) was selected as the stimulation target. The stimulation over right DLPFC (RDLPFC) was designed in only one study (34). Therefore, only the research data of LDLPFC stimulation were included in the pooled estimation.

**Figure 2:**
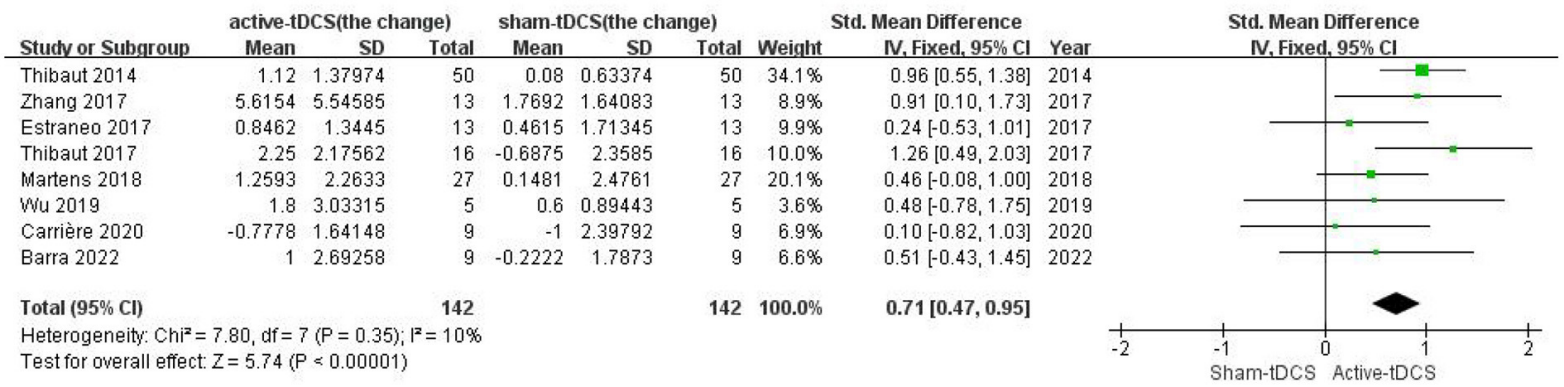
Forest plot for meta-analysis on all studies; SMD (95% CI) between active-tDCS and sham for change of CRS-R score.

**Figure 3:**
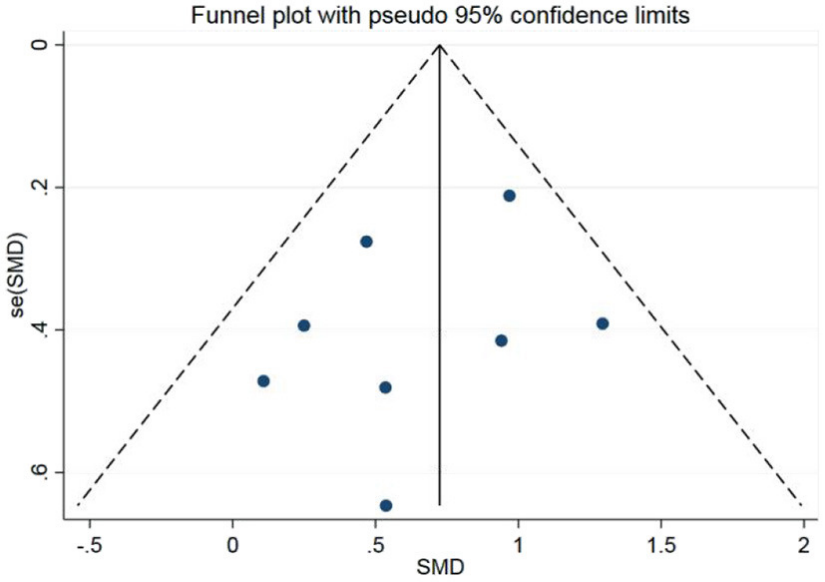
Funnel plot of meta-analysis on all studies.

A fixed-effect model meta-analysis on all studies yielded a significant increase in the CRS-R score after active-LDLPFC-anode-tDCS vs. the sham (SMD = 0.71; 95% CI: 0.47–0.95, *P* < 0.00001; *Chi*^2^ = 7.80, *P* = 0.35, *I*^2^ = 10%; Figure 2), which remained significant in the sensitivity analysis (Table 2). No evidence of publication bias has been found, as indicated by the funnel plot (Figure 3) and Egger's test (*P* = 0.363, Table 3 and Supplementary Figure S3). However, the existing data were insufficient to support a synthesis analysis to estimate the durability of the treatment effect.

**Table 2:** Summary of leave-one-out sensitivity analysis for total studies included in quantitative synthesis (*n* = 8).

**Study omitted**	**95% CI**	**Heterogeneity**			**Effect**		**Weight in total synthesis**
		***Chi^2^***	***P***	***I^2^***	**Z**	***P***	
Thibaut 2014	0.58 [0.28, 0.88]	5.65	0.46	0%	3.80	0.0001	34.1%
Zhang 2017	0.69 [0.44, 0.94]	7.54	0.27	20%	5.33	< 0.00001	8.9%
Estraneo 2017	0.76 [0.51, 1.02]	6.24	0.40	4%	5.84	< 0.00001	9.9%
Thibaut 2017	0.65 [0.39, 0.90]	5.59	0.47	0%	4.97	< 0.00001	10.0%
Martens 2018	0.77 [0.50, 1.04]	5.58	0.34	12%	5.58	< 0.00001	20.1%
Wu 2019	0.72 [0.47, 0.96]	7.68	0.26	22%	5.70	< 0.00001	3.6%
Carrière 2020	0.75 [0.50, 1.01]	6.03	0.42	0%	5.89	< 0.00001	6.9%
Barra 2022	0.72 [0.47, 0.97]	7.62	0.27	21%	5.65	< 0.00001	6.6%
Total	0.71 [0.47, 0.95]	7.80	0.35	10%	5.74	< 0.00001	100.0%

**Table 3:** Egger's test for publication bias of meta-analysis on all studies.

	***Coef*.**	***Str. Err*.**	***t-*value**	***P*-value**	**[95% CI]**
Slope	1.096298	0.4008151	2.74	0.034	[0.1155389, 2.077057]
Bias	−1.127848	1.146478	−0.98	0.363^a^	[−3.93318, 1.677483]

^a^P = 0.363 indicate no publication bias.

### Subgroup analysis—PDOC diagnosis

**Figure 4:**
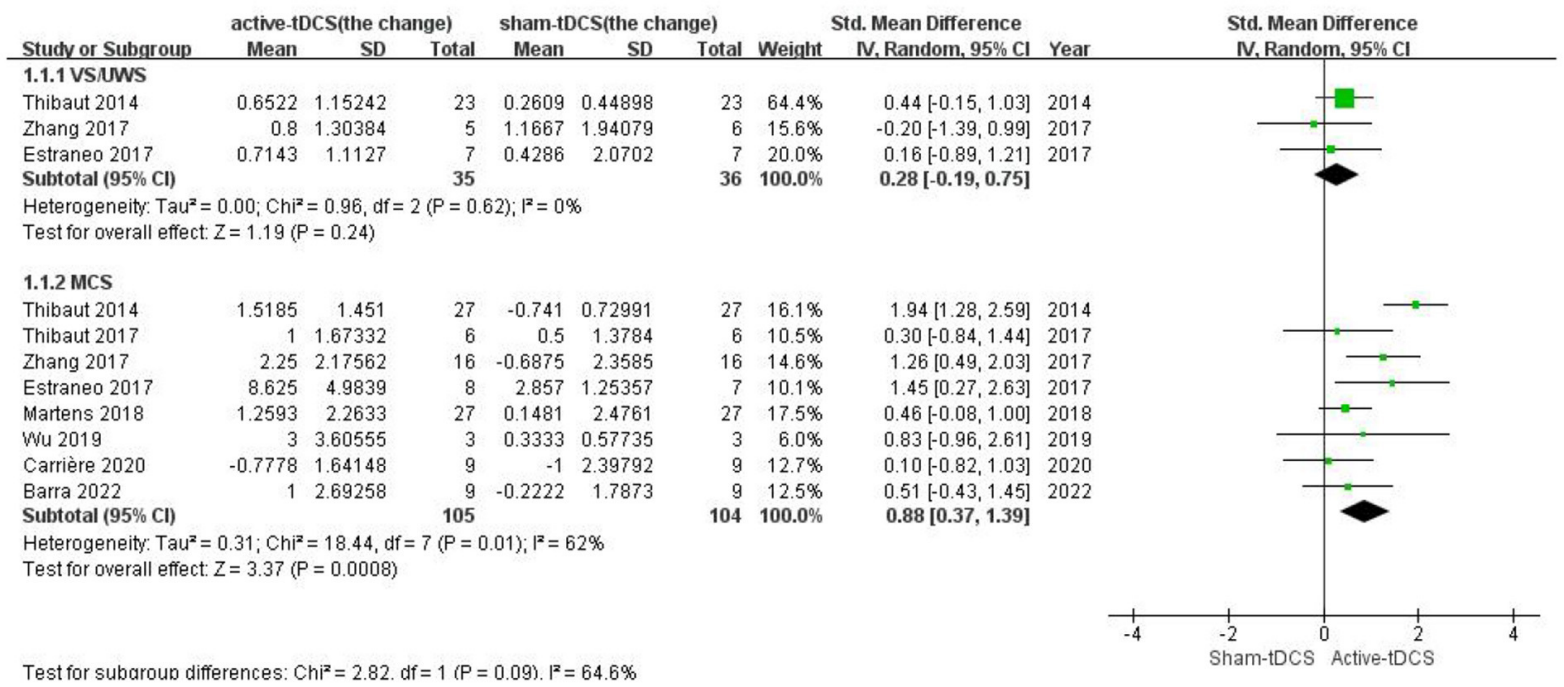
Subgroup analysis of different PDOC diagnoses.

For the patients with MCS, a meta-analysis based on all 8 studies showed a significant effect in favor of active-tDCS compared with the sham intervention (SMD = 0.88; 95% CI: 0.37–1.39, *P* = 0.0008; Figure 4). However, a moderate heterogeneity existed among the studies (*Chi*^2^ = 18.44, *P* = 0.01, *I*^2^ = 62%). A sensitivity analysis indicated that the heterogeneity was only attributed to 1 study (24) in which the rate of individuals who could be initially diagnosed with MCS+ was higher than that in other studies. The better neural structural integrity of patients with MCS+ may make them easier to respond to interventions (10, 35). After the trial was excluded, the heterogeneity disappeared (*Chi*^2^ = 6.48, *P* = 0.37, *I*^2^ = 7%) and the pooled effect remained significant (SMD = 0.65; 95% CI: 0.32–0.97, *P* = 0.0001; Supplementary Figure S4).

For the patients with UWS, only 3 studies with sufficient data were included (24, 31, 32) and reported insignificant effect without heterogeneity (SMD = 0.28; 95% CI: −0.15 to 1.03, *P* = 0.24; *Chi*^2^ = 0.96, *P* = 0.62, *I*^2^ = 0%; Figure 4).

### Subgroup analysis—clinical diagnosis

**Figure 5:**
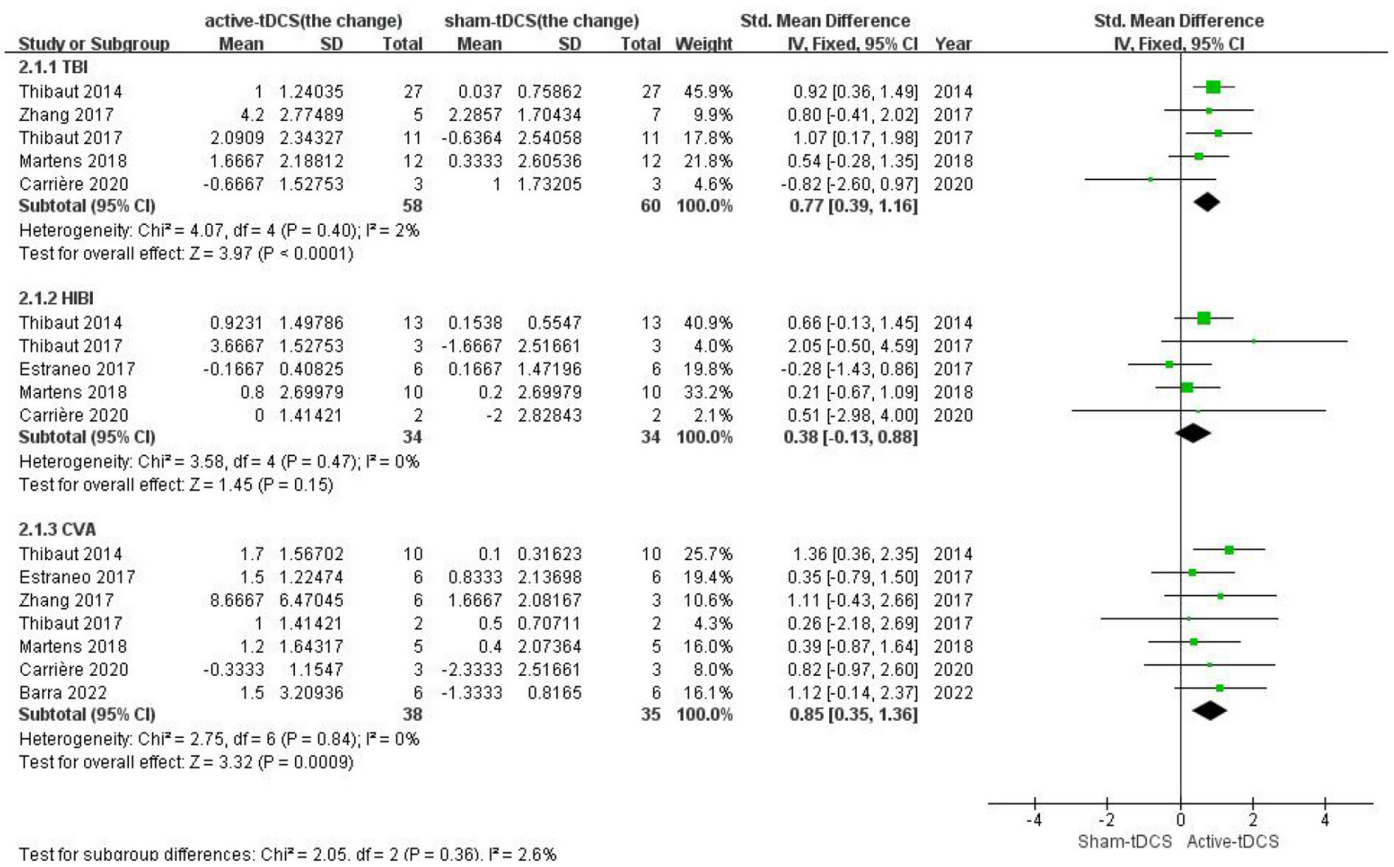
Subgroup analysis of different clinical diagnoses.

For the patients with TBI, five studies with sufficient data were included (24, 29–31, 33). The CRS-R score was significantly increased after active-tDCS compared with the Sham (SMD = 0.77; 95% CI: 0.39–1.16, *P* < 0.0001; *Chi*^2^ = 4.07, *P* = 0.40, *I*^2^ = 2%; Figure 5). As well as for the subgroup of patients with CVA, which included seven studies (24, 25, 29–33), the significant effect was shown (SMD = 0.85; 95% CI: 0.35–1.36, *P* = 0.0009; *Chi*^2^ = 2.75, *P* = 0.84, *I*^2^ = 0%; Figure 5). No significant effect was given on the subgroup of patients with HIBI (SMD = 0.38; 95% CI: −0.13 to 0.88, *P* = 0.15; *Chi*^2^ = 3.58, *P* = 0.47, *I*^2^ = 0%; Figure 5), which included five studies (24, 29, 30, 32, 33). As the heterogeneity was low, a fixed-effects model was used.

We also performed a *Chi*^2^ test to compare the ratio of UWS and MCS in the three clinical diagnoses, and the results showed no statistical difference (*Chi*^2^ = 5.034, *P* = 0.081) (Supplementary Table S3).

### Subgroup analysis—disease course

In the subgroup analysis based on different disease courses, all subgroups showed statistical significance and no heterogeneity. The details are presented in Table 4 and Supplementary Figure S5.

**Table 4:** Subgroup analysis of different disease course.

**Time post-injury^a^**	**Number of studies included**	**Number of individuals involved**	**Effect**			**Heterogeneity**		
			**Z**	**95% CI**	***P***	***Chi^**2**^***	***P***	***I^**2**^***
Disease course ≤ 3 month	5 (24, 25, 29, 31, 32)	32	3.06	0.91 [0.33, 1.50]	= 0.002	5.88	0.21	32%
Disease course >3 month	8 (24, 25, 29–34)	128	4.44	0.61 [0.34, 0.88]	< 0.00001	7.46	0.38	6%
Disease course >6 month	7 (24, 29–34)	106	4.49	0.67 [0.38, 0.96]	< 0.00001	3.14	0.79	0%
Disease course >12 month	6 (24, 29, 30, 32–34)	86	3.46	0.56 [0.24, 0.87]	= 0.0005	0.84	0.97	0%
Disease course >24 month	5 (24, 30, 32–34)	62	3.62	0.71 [0.33, 1.14]	< 0.0003	3.92	0.42	0%
Disease course >36 month	4 (24, 30, 33, 34)	52	3.11	0.67 [0.25, 1.09]	= 0.002	4.08	0.25	26%
Disease course >48 month	4 (24, 30, 33, 34)	42	2.65	0.64 [0.17, 1.12]	= 0.008	5.58	0.13	46%

^a^The subgroup with a longer disease course was not further analyzed for the sample size of patients was too small.

### Descriptive analysis of neuroelectrophysiology findings

Six studies explored the neuroelectrophysiology results by electroencephalogram (EEG) analysis, and one study used event-related potential (ERP). Connectivity, power spectrum, and coherence were common indicators involved. LDLPFC-tDCS tended to augment the connectivity between the frontal parietal lobe and the interhemispheric parietal lobe, especially in theta and alpha bands (25, 28, 29, 34). Similar results also occurred in the delta band and beta band (28, 34). Cavinato et al. (27) found some more complex changes in alpha, delta, and beta bands, including coherence and power spectrum, but no change in theta. Barra et al. (25) considered that their findings could support the theta band connectivity as a biomarker of responders to tDCS. Estraneo et al. (32) failed to find any immediate changes in EEG after stimulation, but they agreed with the idea that quantitative analysis of EEG can be used as a tool to identify intervention responders. P300 amplitude was reported to increase significantly after LDLPFC-tDCS (31). Based on that change, the author pointed out that the improvement in consciousness relevant to tDCS may be related to the improvement in attention resource allocation (31).

Most of the above positive transcerebral changes in EEG and ERP were only found after LDLPFC-tDCS in patients with MCS. Transcerebral significant changes in a wide range were rarely found in patients with UWS after stimulation, and the observable EEG changes were limited in the stimulation site.

## Discussion

In the present study, we synthesize eight articles, involving 165 patients with PDOC (UWS or MCS, with a disease course of more than 28 days). Meta-analysis, including all studies, found that LDLPFC-anode-tDCS had a significant immediate effect on the improvement of CRS-R scores, without depending on the disease course. However, effectiveness could not be demonstrated in patients diagnosed with UWS or HIBI in subgroup analyses.

Compared with the previous analysis of tDCS in a systematic review of all types of NIBS for patients with DOC published in 2020 (21), 2 new studies (25, 29) were added to our synthesis, and a previously incomplete data set (24, 36) (which has the largest sample size of all studies) was supplemented. The total sample size of the participants increased by 68.4%. The two new studies remain controversial in terms of tDCS improving the CRS-R score. However, the main result of our analysis is similar to those before. This finding could indicate that LDLPFC-anode-tDCS could have a positive therapeutic effect on patients with MCS but an insignificant effect on patients with UWS. This finding is consistent with the common recognition that MCS has a better prognosis than UWS (37).

As the recent tendency seems to favor the use of the mesocircuit model to directly explore DOC (38), which may lead to ignoring differences caused by diverse clinical diagnoses, we added it to the subgroup analysis. From the available data, we divided the diagnoses into CVA (including cerebral hemorrhage, subarachnoid hemorrhage, ischemic stroke, etc.), TBI, and HIBI (including hypoxia, anoxic and cardiac arrest, etc.) (39, 40) to conduct subgroup analysis. Only the HIBI group did not show sufficient sensitivity to LDLPFC-tDCS by assessment with the CRS-R score. Given the complexity of injury mechanisms in HIBI (39, 41), these patients are generally considered more difficult to recover (33). Unfortunately, in our analysis, the inferior prognosis of HIBI compared with TBI or CVA failed to be compensated by tDCS stimulus. Interestingly, compared with the two other diagnostic subgroups, the HIBI group did not show a significant disadvantage in the ratio of UWS or baseline CRS-R score (Supplementary Table S3), while the responses of stimulation were generally considered to be related to the baseline CRS-R score (42) and the type of PDOC (Figure 4). This could mean that the existing behavioral tests and PDOC classification methods still have defects in predicting the prognosis of patients with PDOC (43). Though the EEG or other multi-modal neurological examination could partly help to evaluate the residual brain function and predict the prognosis (44), the specificity and uniformity of the standards of these methods were far from reaching routine clinical application. The neurological function test of patients with PDOC is rarely conducted in accordance with the diagnosis of primary diseases. This might be a study perspective in the future.

The result can also indirectly reflect that the mesocircuit model may have not been fully clarified and is insufficient to explain all the mechanisms of PDOC (45). According to the model, the best target of tDCS or other NIBS with a shallow effective depth seems to be naturally concentrated in DLPFC; the excitability from the prefrontal to the striatum was believed to be beneficial to disinhibit the pallidum to the central thalamus (1, 12). Stimulation over LDLPFC could mediate functional connectivity at the distant regions of the neural network (46). The stimulation effect on the other parts in the model (such as the posterior parietal cortex) was considered worse than that on DLPFC (47). However, the controversy remains. Some studies (48, 49) have shown that the value of tDCS over primary motor cortex (M1) deserves further exploration. Some researchers also consider the cerebellum to be related to the mesocircuit model (50). Aloi et al. (14) suggested that the cathodal-cerebellum combined with anodal-M1 could be regarded as a potential tDCS montage for the treatment of PDOC, while the cerebellum and M1 have not yet been included when describing the mesocircuit model. In addition, compared with the single-target stimulation protocol commonly used in scientific research, from the perspective of clinical practice, Zhang et al. (51) believe that multi-target stimulation will be more practical and effective. However, as our literature search strategy did not limit the stimulation target, the retrieval results could show that the existing evidence of other targets is insufficient to sustain a high-quality synthesis. Further research about the diversity of stimulation targets should be performed to provide evidence for improving the model mesocircuit model.

It is still controversial whether the disease course of PDOC patients will affect the treatment efficacy. The previous evidence does not clearly support or refute the time post-injury as a prognostic factor (2). Some researchers have suggested that different disease courses may lead to different stimulation effects (31). But long-term and repeated treatment is necessary for individuals with PDOC (51, 52), and from the perspective of avoiding the self-fulfilling prophecy (41, 53), it seems reasonable to weaken the influence of the disease course when dealing with patients with PDOC. We conducted the subgroup analysis of different disease courses and the result could provide evidence for the exclusion of time post-injury from prognostic indicators.

Finally, the safety of tDCS was proven in the studies included in our review. No adverse effects were reported to be associated with tDCS, although dropouts existed. The reasons for all dropouts were as follows: 6 withdrew because of infections (27, 30, 31), 6 withdrew because of unplanned treatment modifications (25, 33), 1 withdrew due to transportation issue (33), and 7 withdrew because they failed to complete the behavioral assessment, which was delayed by other clinical affairs (29, 30), and 3 withdrew because of severe adverse events (death, seizures, etc.) for other medical reasons external to these studies (25, 30, 33). According to the current evidence provided by our review, in the absence of sufficient effective treatments for PDOC, LDLPFC-tDCS can be considered as a feasible clinical option to improve awareness in individuals with PDOC considering the safety and the certain treatment effect on some populations with specific diagnosis.

## Study limitations

This study has three main limitations. First, we only analyzed the short-term effect of the intervention. We intended to evaluate the long-term effect by changing between the follow-up data and the data immediately after the intervention. Most of the included studies lacked follow-up data, which makes it impossible to complete the test of the long-term effect. Second, although the funnel plot and Egger's test showed no publication bias, their results may still be subject to some constraints due to the limited total sample size and the relative concentration of published countries. Finally, although we have made a descriptive summary of the results of neuroelectrophysiology tests, data synthesis and meta-analysis are difficult to conduct, due to no unified standard for the types, paradigm, and observation indicators of these neuroelectrophysiology tests on PDOC.

## Conclusions

In conclusion, anodal-tDCS over the left DLPFC may be advantageous to the recovery of patients with MCS and clinically diagnosed with CVA or TBI. No evidence can support that duration of the disease course will limit the performance of the treatment effect. Further studies are needed to explore the diversity of stimulation targets.

## Data availability statement

The original contributions presented in the study are included in the article/Supplementary material, further inquiries can be directed to the corresponding author.

## Author contributions

SL, QG, and MG: concept, idea, and research design. SL and YC: writing. WM and SC: literature search. CL and BL: data extraction. SL and SC: data analysis. SL, YC, and QG: project management. SL, QG, and LY: consultation including review of manuscript before submitting. All authors contributed to manuscript revision, read, and approved the submitted version.

## Funding

This study was supported by NSFC 82172540 from the National Natural Science Foundation of China.

## Conflict of interest

The authors declare that the research was conducted in the absence of any commercial or financial relationships that could be construed as a potential conflict of interest.

## Publisher's note

All claims expressed in this article are solely those of the authors and do not necessarily represent those of their affiliated organizations, or those of the publisher, the editors and the reviewers. Any product that may be evaluated in this article, or claim that may be made by its manufacturer, is not guaranteed or endorsed by the publisher.
