# Supplementary material for: Effectiveness of transcranial direct current stimulation over dorsolateral prefrontal cortex in patients with prolonged disorders of consciousness: A systematic review and meta-analysis
Source: Front Neurol. 2022 Sep 26;13:998953. doi: 10.3389/fneur.2022.998953 (PMC9549167; doi:10.3389/fneur.2022.998953)
Supplement: Supplementary file 1 [file Table_1.DOCX]

**The examples of Search Strategy**

Database: MEDLINE (via PubMed,from the establishment to March 26, 2022).

Search string: ((tDCS) OR (transcranial direct current stimulation)) AND ((MCS) OR (minimally conscious state) OR (disorder of consciousness) OR (coma) OR (unresponsive wakefulness syndrome) OR (vegetative state) OR (disturbance of consciousness)).

Processed as follow:

*#1 Search: Transcranial direct current stimulation*

*#2 Search: tDCS*

*#3 Search: #1 OR #2*

*#4 Search: MCS*

*#5 Search: minimally conscious state*

*#6 Search: disorder of consciousness*

*#7 Search: coma*

*#8 Search: unresponsive wakefulness syndrome*

*#9 Search: vegetative state*

*#10 Search: disturbance of consciousness*

*#11 Search: #4 OR #5 OR #6 OR #7 OR #8 OR #9 OR #10*

*#12 Search: #3 AND #11*

Database: Embase (from 1974 to 2022 March 26).

*#1*: Transcranial direct current stimulation.mp. [mp=title, abstract, heading word, drug trade name, original title, device manufacturer, drug manufacturer, device trade name, keyword heading word, floating subheading word, candidate term word] 9800

*#2*: tDCS.mp. [mp=title, abstract, heading word, drug trade name, original title, device manufacturer, drug manufacturer, device trade name, keyword heading word, floating subheading word, candidate term word]

*#3*: 1 or 2

*#4*: MCS.mp. [mp=title, abstract, heading word, drug trade name, original title, device manufacturer, drug manufacturer, device trade name, keyword heading word, floating subheading word, candidate term word]

*#5*: minimally conscious state.mp. [mp=title, abstract, heading word, drug trade name, original title, device manufacturer, drug manufacturer, device trade name, keyword heading word, floating subheading word, candidate term word]

*#6*: disorder of consciousness.mp. [mp=title, abstract, heading word, drug trade name, original title, device manufacturer, drug manufacturer, device trade name, keyword heading word, floating subheading word, candidate term word]

*#7*: coma.mp. [mp=title, abstract, heading word, drug trade name, original title, device manufacturer, drug manufacturer, device trade name, keyword heading word, floating subheading word, candidate term word]

*#8*: unresponsive wakefulness syndrome.mp. [mp=title, abstract, heading word, drug trade name, original title, device manufacturer, drug manufacturer, device trade name, keyword heading word, floating subheading word, candidate term word]

*#9*: Vegetative state.mp. [mp=title, abstract, heading word, drug trade name, original title, device manufacturer, drug manufacturer, device trade name, keyword heading word, floating subheading word, candidate term word]

*#10*: Disturbance of consciousness.mp. [mp=title, abstract, heading word, drug trade name, original title, device manufacturer, drug manufacturer, device trade name, keyword heading word, floating subheading word, candidate term word]

*#11*: 4 or 5 or 6 or 7 or 8 or 9 or 10

*#12*: 3 and 11

EBM Reviews - Cochrane Central Register of Controlled Trials <January 2022>

1 Disturbance of consciousness.mp. [mp=title, original title, abstract, mesh headings, heading words, keyword] 79

2 Vegetative state.mp. [mp=title, original title, abstract, mesh headings, heading words, keyword] 234

3 unresponsive wakefulness syndrome.mp. [mp=title, original title, abstract, mesh headings, heading words, keyword] 26

4 coma.mp. [mp=title, original title, abstract, mesh headings, heading words, keyword] 3984

5 disorder of consciousness.mp. [mp=title, original title, abstract, mesh headings, heading words, keyword] 38

6 minimally conscious state.mp. [mp=title, original title, abstract, mesh headings, heading words, keyword] 113

7 MCS.mp. [mp=title, original title, abstract, mesh headings, heading words, keyword] 1703

8 1 or 2 or 3 or 4 or 5 or 6 or 7 5826

9 Transcranial direct current stimulation.mp. [mp=title, original title, abstract, mesh headings, heading words, keyword] 4682

10 tDCS.mp. [mp=title, original title, abstract, mesh headings, heading words, keyword] 4456

11 9 or 10 5138

12 8 and 11 65
