# Supplementary material for: Effectiveness of transcranial direct current stimulation over dorsolateral prefrontal cortex in patients with prolonged disorders of consciousness: A systematic review and meta-analysis
Source: Front Neurol. 2022 Sep 26;13:998953. doi: 10.3389/fneur.2022.998953 (PMC9549167; doi:10.3389/fneur.2022.998953)
Supplement: Supplementary file 2 [file Table_2.DOCX]

**Supplementary Table 1.** Summary of PEDro criteria for included studies (n= 10)

| Study  (year) | PEDro Scale Items^a^ | | | | | | | | | | PEDro  Score (0-10) | RCT  quality ^b^ |
| --- | --- | --- | --- | --- | --- | --- | --- | --- | --- | --- | --- | --- |
|  | 2 | 3 | 4 | 5 | 6 | 7 | 8 | 9 | 10 | 11 |  |  |
| Thibaut 2014 | Y | Y | Y | Y | N | Y | Y | N | Y | Y | 8 | good |
| Thibaut 2017 | Y | Y | Y | Y | N | Y | N | N | Y | Y | 7 | good |
| Zhang 2017 | Y | N | Y | Y | N | Y | Y | N | Y | Y | 7 | good |
| Estraneo 2017 | Y | N | Y | Y | N | Y | Y | N | Y | Y | 7 | good |
| Martens 2018 | Y | Y | Y | Y | N | Y | N | Y | Y | Y | 8 | good |
| Wu 2019 | Y | N | Y | Y | N | Y | Y | N | Y | Y | 7 | good |
| Carrière 2020 | Y | N | Y | Y | N | Y | N | N | Y | Y | 6 | good |
| Barra 2022 | Y | Y | Y | Y | N | Y | Y | N | Y | Y | 8 | good |
| Cavinato 2019 | Y | N | Y | Y | N | Y | Y | N | Y | Y | 7 | good |
| Thibaut 2019 | Y | N | Y | Y | Y | Y | Y | N | Y | Y | 8 | good |

Y: yes; N: no.

^a^ 2: random allocation; 3: concealed allocation; 4: baseline comparability; 5: blinded participants; 6: blinded therapists; 7: blind assessors; 8: adequate follow-up; 9: intention-to-treat analysis; 10: between-group comparisons; 11: point estimates and variability.

^b^ It is considered that the scores less than 4 are ‘poor’, 4 to 5 are ‘fair’, 6 to 8 are ‘good’ and 9 to 10 are ‘excellent’.

**Supplementary Table 2：**The quality of evidence based on GRADE criteria. (**Question:** Active-DLPFC-anode-tDCS compared to Sham-tDCS for individuals with PDOC)

| **Quality assessment** | | | | | | | | **No of patients** | | **Effect** | | **Quality** | **Importance** | |
| --- | --- | --- | --- | --- | --- | --- | --- | --- | --- | --- | --- | --- | --- | --- |
|  |  |  |  |  |  |  |  |  |  |  |  |  |  |  |
| **No of studies** | | **Design** | **Risk of bias** | **Inconsistency** | **Indirectness** | **Imprecision** | **Other considerations** | **Active-tDCS** | **Sham-tDCS** | **Relative**  **(95% CI)** | **Absolute** |  |  |  |
| **change of CRS-R score (Better indicated by higher values)** | | | | | | | | | | | | | | |
| 8 | | parallel or cross-over randomized controlled trials | no serious risk of bias | no serious inconsistency | no serious indirectness | Serious^1^ | none | 142 | 142 | - | SMD 0.71 higher (0.47 to 0.95 higher) | ⊕⊕⊕O  MODERATE | - | |
| ^1^**Downgraded one level for serious imprecision:**total population size is less than 400 (as rules-of-thumb value suggested for continuous variable)  **SMD:** Standardized mean difference; **CI:** Confidence interval; | | | | | | | | | | | | |  |  |
| GRADE Working Group grades of evidence：  **High quality:** Further research is very unlikely to change our confidence in the estimate of effect.  **Moderate quality:** Further research is likely to have an important impact on our confidence in the estimate of effect and may change the estimate.  **Low quality:** Further research is very likely to have an important impact on our confidence in the estimate of effect and is likely to change the estimate.  **Very low quality:** We are very uncertain about the estimate. | | | | | | | | | | | | |  |  |

**Supplementary Table 3.** Characteristics of patients with different clinical diagnoses involved in subgroup analysis

| Subgroup of clinical diagnostics | Gender | | | | PDOC classification^a^ | | | | CRS-R before active-tDCS^b^ | | |
| --- | --- | --- | --- | --- | --- | --- | --- | --- | --- | --- | --- |
|  | M | F | *Chi^2^* | *P* | MCS | UWS | *Chi^2^* | *P* | Median (quartile) | Compared with HIBI | |
|  |  |  |  |  |  |  |  |  |  | *Z* | *P* |
| CVA (n=41) | 21 | 20 | 4.344 | 0.114 | 28 | 13 | 5.034 | 0.081 | 7 (4, 9) | -0.326 | 0.744 |
| TBI (n=65) | 43 | 22 |  |  | 54 | 11 |  |  | 9 (6, 11.5) | -2.343 | 0.019 |
| HIBI (n=34) | 25 | 9 |  |  | 22 | 12 |  |  | 6.5 (4.75, 9.25) | / | |

^a^: The *Chi^2^* test indicates that there is no significant disadvantage in the DOC classification (*P*＞0.05)

^b^: The U-test was used to compare the differences between HIBI group and other two groups. The baseline CRS-R score of HIBI group was significantly lower than that of TBI group, but there was no significant difference with that of CVA group (*P*＞0.05). It indicates that there is no significant disadvantage in the baseline CRS-R score of the HIBI group vs CVA group.
