# Supplementary figures and images for: Effectiveness of transcranial direct current stimulation over dorsolateral prefrontal cortex in patients with prolonged disorders of consciousness: A systematic review and meta-analysis
Source: Front Neurol. 2022 Sep 26;13:998953. doi: 10.3389/fneur.2022.998953 (PMC9549167; doi:10.3389/fneur.2022.998953)

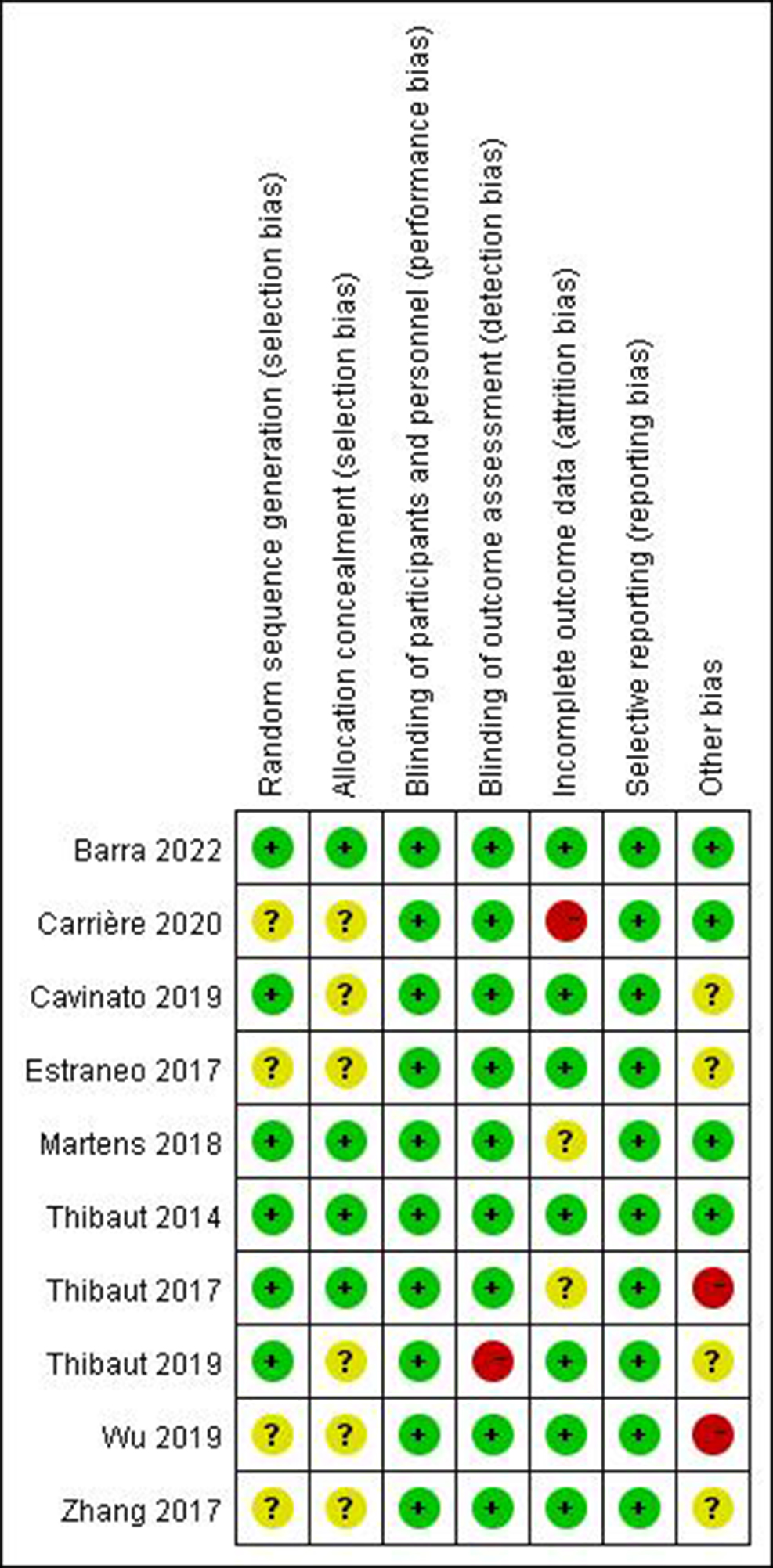

Supplement: Supplementary file 3 [file Image_1.JPEG]

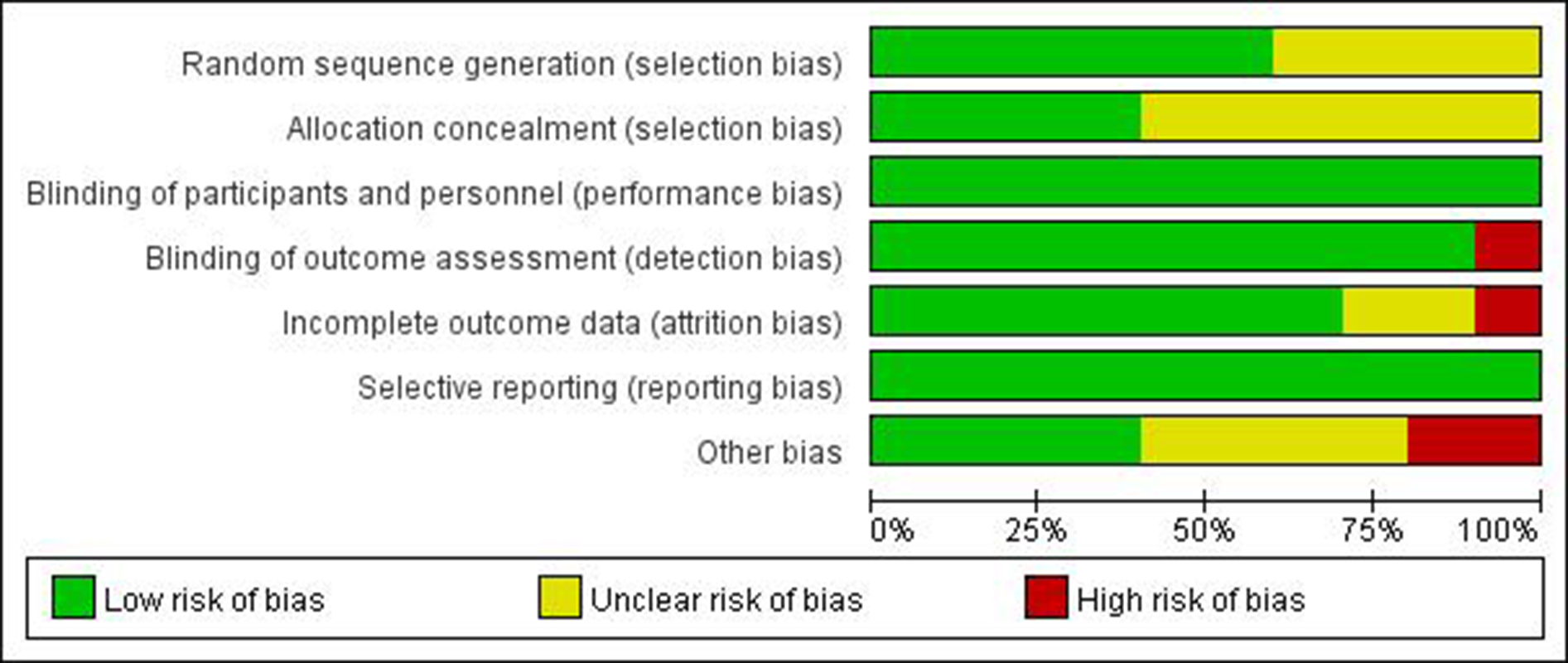

Supplement: Supplementary file 4 [file Image_2.JPEG]

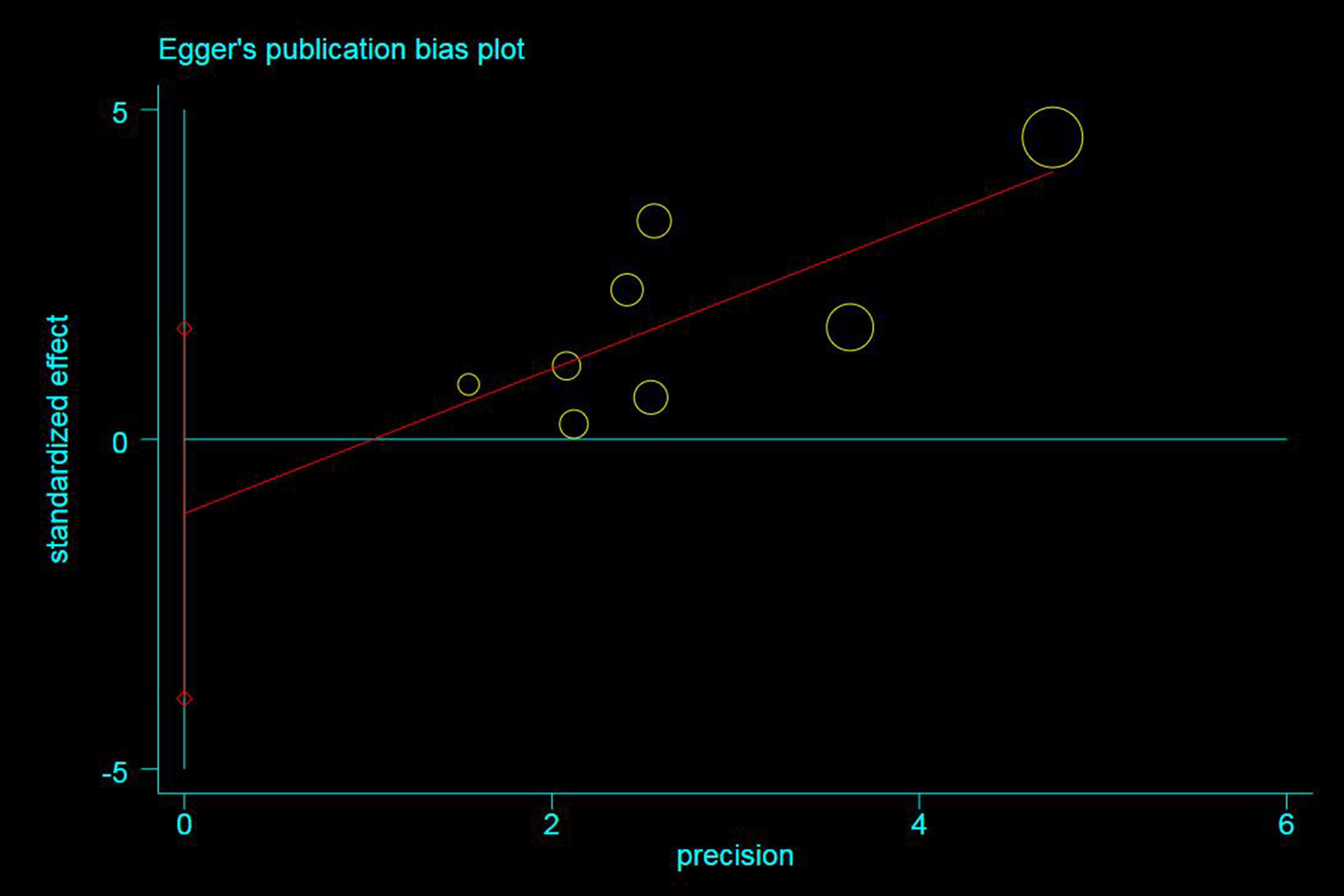

Supplement: Supplementary file 5 [file Image_3.JPEG]

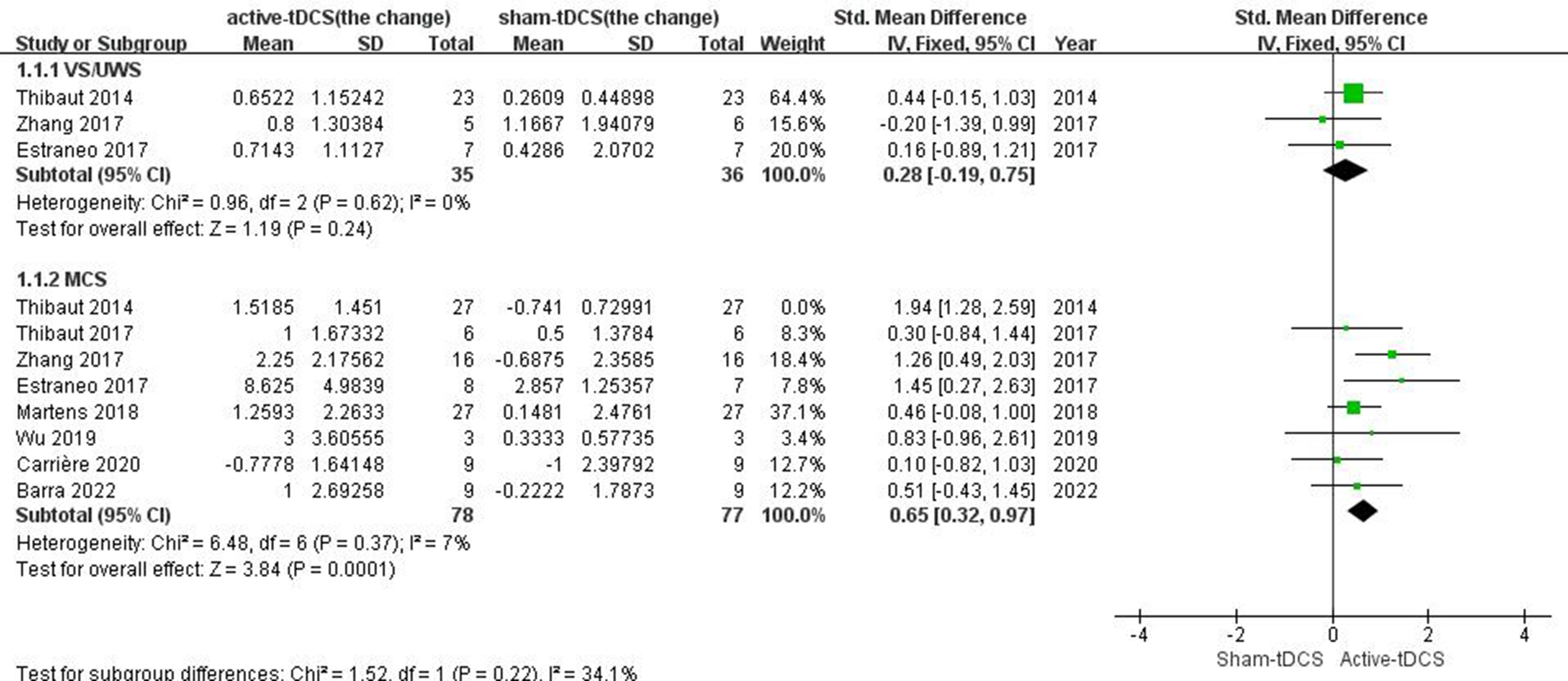

Supplement: Supplementary file 6 [file Image_4.JPEG]

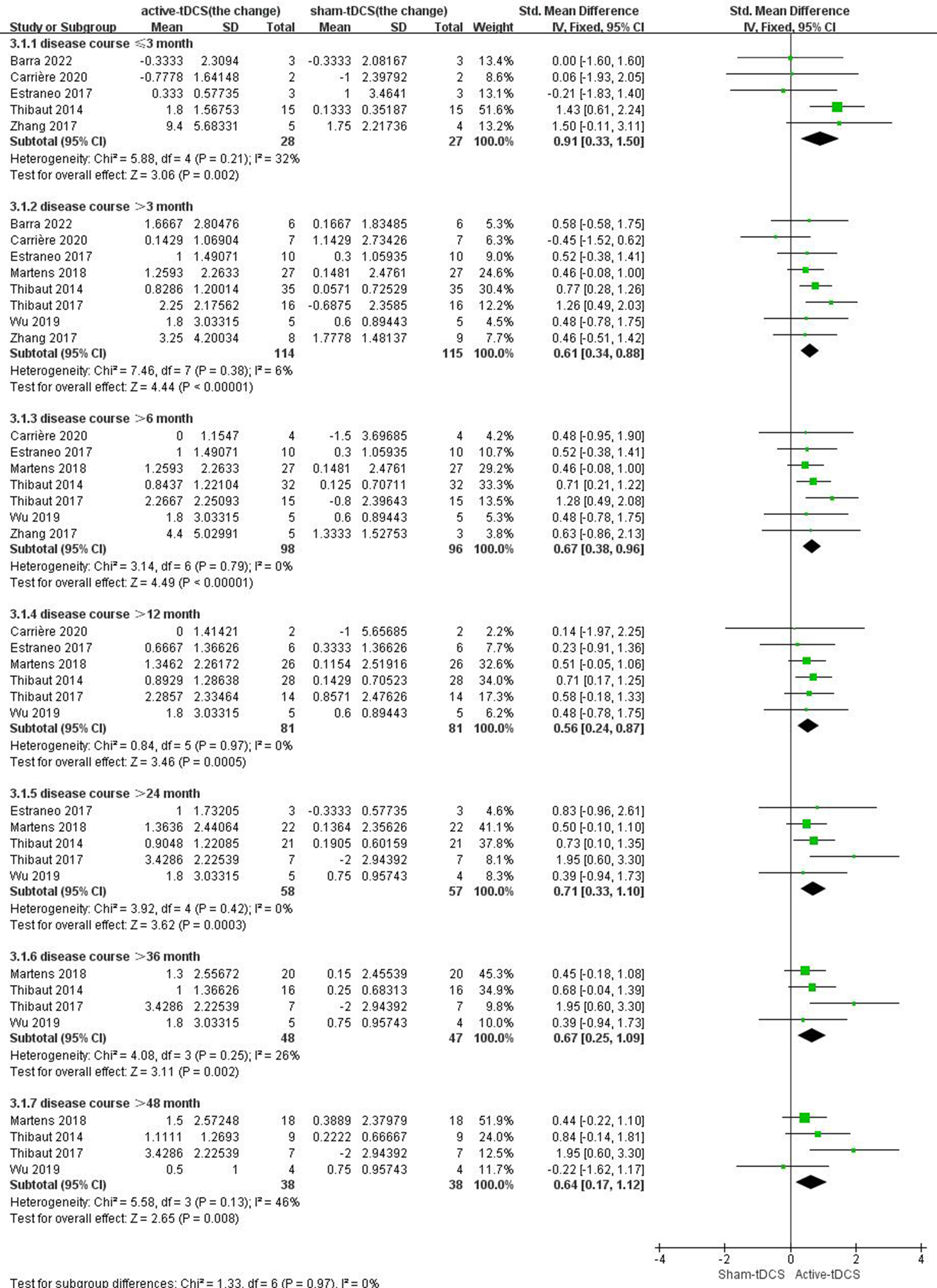

Supplement: Supplementary file 7 [file Image_5.JPEG]
